# Genome display tool: visualizing features in complex data sets

**DOI:** 10.1186/1751-0473-2-1

**Published:** 2007-02-14

**Authors:** Lalitha Viswanath, Yue Lu, George E Fox

**Affiliations:** 1 Department of Biology and Biochemistry, 3201 Cullen Boulevard, University of Houston, Houston, Texas 77204-5001, USA; 2 University of Maryland Biotechnology Institute, 9600, Gudelsky Drive, Rockville, MD 20850, USA; 3 Department of Epidemiology, 1515 Holcombe Blvd, M.D. Anderson Cancer Centre, Houston, TX 77030, USA

## Abstract

**Background:**

The enormity of the information contained in large data sets makes it difficult to develop intuitive understanding. It would be useful to have software that allows visualization of possible correlations between properties that can be associated with a core data set. In the case of bacterial genomes, existing visualization tools focus on either global properties such as variations in composition or detailed local displays of the features that comprise the annotation. It is not easy to visualize other information in the context of this core information.

**Results:**

A Java based software known as the Genome Display Tool (GDT), allows the user to simultaneously view the distribution of multiple attributes pertaining to genes and intragenic regions in a single bacterial genome using different colours and shapes on a single screen. The display represents each gene by small boxes that correlate with physical position in the genome. The size of the boxes is dynamically allocated based on the number of genes and a zoom feature allows close-up inspection of regions of interest. The display is interfaced with a MS-Access relational database and can display any feature in the database that can be represented by discrete values. Data is readily added to the database from an MS-Excel spread sheet. The functionality of GDT is demonstrated by comparing the results of two predictions of recent horizontal transfer events in the genome of *Synechocystis *PCC-6803. The resulting display allows the user to immediately see how much agreement exists between the two methods and also visualize how genes in various categories (e.g. predicted in both methods, one method etc) are distributed in the genome.

**Conclusion:**

The GDT software provides the user with a powerful tool that allows development of an intuitive understanding of the relative distribution of features in a large data set. As additional features are added to the data set, the number of possible correlations that can be visualized grows rapidly. Although described here for use in bacterial genomics, the principle is general and similar software might be useful in other contexts such as patient studies.

## Background

A key step in the creative process is intuition. However, the enormity of the information contained in large data sets frequently makes it difficult to develop the needed intuitive understanding of the properties of the data. It is useful to have tools that allow visualization of possible relationships between properties that can be associated with the core data set. In the case of bacterial genomes, existing visualization tools focus on either global properties such as variations in composition throughout the genome or very detailed local displays of features that comprise the genome annotation. For example, the commercially available GenVision [1] allows easy construction of circular diagrams that allow the tracking of properties such as GC content, gene orientation, etc. on a global scale. At higher resolution, GenVision can compare gene order in local regions of multiple genomes or various features of the detailed annotation in local regions of a single genome. Annotation systems such as GBrowse [2] and Artemis [3] likewise facilitate both annotation and very sophisticated viewing of the features added to the annotation file with the ability to zoom in and out. However, the amount of information included precludes an easy examination of the whole genome at once on a single screen. Genboree [4] provides a variety of comparison capabilities for eukaryotic genomes and again has at its core the ability to zoom in and out of a visualization in which genes are represented to scale with orientation shown. A custom track option has recently been added to the UCSC genome browser [5,6] that allows the user to add non-standard information as a separate track that is displayed along with the usual annotation data. Despite the sophistication of these and other tools, it is not easy to usefully visualize user specific information in the context of the whole genome. Thus, if one suspected that genes predicted to be laterally transferred were clustered in the genome this would not be readily visualized. To address this type of problem, there is a need for software that operates at intermediate levels of resolution that can flexibly incorporate additional user provided information in the context of the core annotation data.

Our efforts to accomplish this purpose were inspired by the dynamic COG (Clusters of Orthologous Groups) [7] tables that are used on the NCBI website [8]. These tables represent each gene in the genome as a colored box in a matrix where the choice of color indicates the functional family that the gene belongs to, if that is known. By clicking on individual boxes, one can drill down to a detailed map of the relevant region of the genome and from there to even more detailed information. Such COG tables are available for many bacterial genomes on the NCBI site including *Escherichia coli *K12 [9]. The GDT software described here uses essentially the same display. The primary difference is that the user is able to control the features that are displayed by the individual boxes in the grid. The purpose of the GDT software is to allow the user to think about the properties of genes in a genome wide context. To facilitate this, GDT allows the user to simultaneously view the occurrence of multiple biological features relating to a bacterial genome using different colours and shapes.

## Implementation

The GDT software was developed using the Java 2 Platform, Standard Edition Software Development Kit Version 1.5.2, and NetBeans Integrated Development Environment installed on a computer system with Intel x86 processor and 256 mega bytes of Random Access Memory, using Windows XP as the operating system. GDT requires Java 2 Run-time Environment Version 1.4.2 or higher for efficient performance. Since GDT interfaces with a Microsoft Access Database, it requires MS-Access 2003, a part of the Microsoft Office 2003 Professional Edition, to be installed. At least 18 MB of hard disk space is recommended to run the program.

Java is freely available for download [10]. It is an object oriented programming language that can be used to develop powerful, software applications, portable across platforms. The Java code is run by Java Virtual Machine (JVM), instead of by the native operating system. Hence any computer with Java VM installed can run a Java program. This makes the program independent of the computer system on which the application was developed. The Java platform is a software only platform that runs on hardware-based platforms.

GDT uses AWT (Abstract Window Toolkit) and Java Swing, a part of the Java Foundation Classes, to display the genes in a graphical manner. Use of AWT ensures that the application can be easily integrated with a webpage, without requiring the Java Plug-in. The software ensures that only attributes possessing less than thousand unique values can be displayed, to ensure smoother interactivity. The tab-delimited text file created by the user, using Genome Display Tool, can be read into the Access Database and later visualized using Genome Display Tool, if required. A user's manual is available at the project site.

## Results and discussion

The purpose of the GDT software is to allow the user to think about the properties of genes in a genome wide context. To facilitate this GDT allows the user to simultaneously view the occurrence of multiple user provided features relating to a bacterial genome using different colours and shapes. The software merges and overlaps colours and shapes for genes that meet multiple criteria. The ability to merge rather than replace, colours and shapes enables the user to instantly locate genes or proteins with shared attributes relative to their physical location in the genome or data set. An optional simultaneous display of gene orientation is also available for use when transcription direction is of interest. GDT displays the entire set of genes or proteins, in their precise order of occurrence in the genome, in a multi-row rectangular grid in one screen.

GDT is distinct from most other display tools in that it allows the user to view the distribution of attributes without restriction to annotation data alone. Additionally GDT does not impose a restriction on the data format or acceptable inputs thereby offering greater flexibility in visualizing different kinds of data. The user is essentially unrestricted in the features that can be added to the underlying database and can visualize possible correlations between any features that are included in the database. Thus, as new features are added to the underlying database, the number of correlations that can be visually examined increases dramatically.

### Display of genes

The GDT software displays the set of genes or proteins as a multi-row rectangular grid. Each gene is represented by a small box. The genes are arranged on the grid in the precise order in which they appear in the genome. The number of genes displayed in each row depends on the number of genes or proteins to be displayed. The number is small enough to ensure that each gene is displayed with sufficient width and height to ensure clarity, and large enough to ensure that all the genes or proteins are displayed on the screen without the user having to scroll through numerous screens. When the user wishes to view upstream and downstream information, each box is further divided into three smaller boxes, with the first and third being slightly smaller than the second. The first box is used to display features corresponding to the upstream region of the gene. The second box corresponds to the individual gene. The third box is used to display features corresponding to the downstream region of the gene. This feature of displaying the entire set of genes or proteins in one screen eliminates the need to scroll through multiple screens and also provides the user an immediate overview of the distribution of features in the genome.

The user controls which attribute or attributes are displayed for each of the three boxes through a separate menu for each region. This choice is accompanied by the opportunity to select which of over 256 colours and 4 shapes will be associated with each attribute. The directions of the genes located on the forward strand are optionally marked with '+' in the middle box. The user can disable the display of directions in the dataset, if required. In order to display multiple attributes simultaneously, the software mixes colours to give a new, different colour to those genes which possess the same combination of attributes.

### Example application

**Figure 1:**
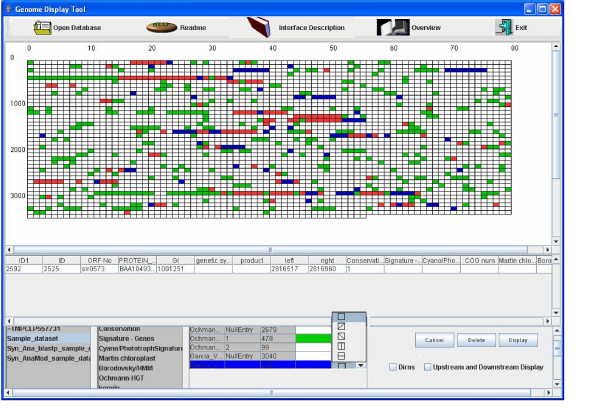
**Genome Display Tool**. The figure demonstrates some of the features of Genome Display Tool, described earlier. The green coloured cells represent the horizontal gene transfer events identified by Ochman et al [11] whereas the blue coloured cells represent the horizontal gene transfer events identified by Garcia and colleagues [12]. The red coloured cells are the genes which are predicted to have been horizontally transferred by both groups. The figure shows the genes on the forward strand with a '+' across them. It also shows the choice of five shapes available to the user to colour each cell. The user can choose to view information in the upstream or downstream region of the gene, if required.

In order to illustrate the utility of GDT, we used it to compare the results of two different predictions of recent horizontal gene transfer events in the genome of *Synechocystis *PCC-6803 [11], Figure 1. The predictions of Ochman *et al*., (2000) [12] were originally coloured green, while those identified by Garcia-Vallve (2000) [13] were coloured blue. The red coloured cells result from the RGB sum of green and blue and represent the genes that were predicted to have been horizontally transferred by both research groups. This ability of the software to merge colours, allows the user to rapidly focus on multiple attributes and display interesting combinations of attributes. Once the user has identified an interesting set of genes, e.g. in the example the genes predicted by both approaches or perhaps the genes missed by one of the approaches the user can write out a file of the genes of interest by choosing *Write information to file*. A text file is then created in tab-delimited format, which can later be imported into a new database or the original database, for further use and visualization.

### Display of additional data from the database

The GDT software interacts with an MS-Access™ database to store the tables containing the data to be viewed. New data can be readily imported into Access from a MS-Excel spread sheet. On selecting a particular table, a list containing all the discrete attributes in the table is shown.

Sometimes, in a gene or protein of interest, it is essential to view additional data apart from the displayed attributes. The software can retrieve such information by connecting to the database, when the user simply clicks on the gene or protein. This additional data is displayed in a separate panel below the graphical display to reduce interference with the graphical display.

By clicking on any single gene on the display the user can obtain additional data from the database corresponding to any gene in a separate panel below the display. Apart from the name of the attribute, the unique values corresponding to the attribute along with the number of genes or proteins possessing that value are also displayed as shown in Figure 1.

### Zoom functionality

**Figure 2:**
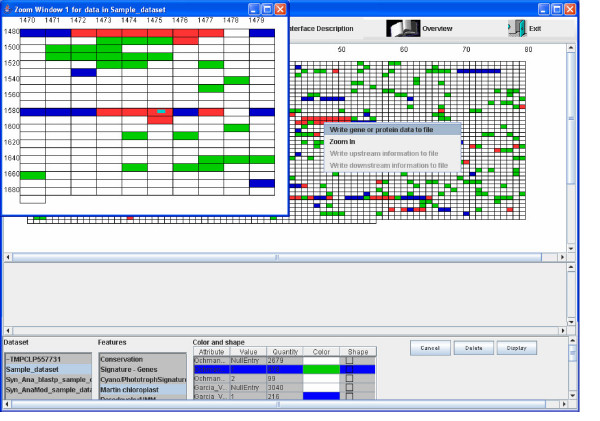
**Zoom Feature in Genome Display Tool**. Figure 2 displays the zoom functionality available with Genome Display Tool. On right-clicking on any gene, the user is presented with the choice of zooming into that region of the genome or, writing out information matching that of the particular cell, its upstream region or its downstream region into a tab-delimited text file. Based on whether the user chose to view interesting information in the upstream or downstream region of the gene, and whether such information is present for the gene in consideration, the latter two choices are enabled or disabled. The zoom functionality results in a separate window showing the region of interest in higher magnification, while retaining the larger display, as shown.

The software allows the user to zoom into any region of the display and view that region in greater detail. The user can click the right button of the mouse when the mouse is over a cell displaying the gene or protein of interest and choose Zoom. This displays 100 genes or proteins upstream and 100 genes or proteins downstream of the gene or protein on which the mouse was clicked, in a new window as shown in Figure 2. The cell which was clicked on is identified in the smaller display by a smaller, filled rectangle in it, as shown in Figure 2.

### Additional comments

The GDT software is very flexible and the user can find a surprisingly large variety of ways to harness its inherent utility. For example, continuous information such as expression data obtained from arrays can be converted to discrete values by assigning ranges of values to discrete groups. In some cases, multiple related attributes exist. For example, the *Synechocystis *PCC-6803 genome has multiple transposases belonging to distinct homology groups. In such a case, one can display each family separately or by simply assigning the same colour to all the individual families display all (or various subsets) of the transposases at once. In addition, database growth provides unexpected flexibility. As one adds new discrete value data to the database one can now correlate the new data with any of the older entries thereby allowing many novel comparisons to be performed.

## Conclusion

GDT is a useful utility for examining various types of data in the context of a whole bacterial genome. This is possible because a modest resolution display is used that allows the entire genome and the data of interest to be visualized on a single screen. Although the software was specifically designed for use with bacterial genomes, it can also be used to display data relating to groups of genes or proteins, e.g. the sporulation genes of *Bacillus*, or groups of proteins. Even more generally it might be used with medical data where each box representing a patient, perhaps progressing from youngest to oldest.

In its current form GDT utilizes platforms, e.g. Windows™ and software, Microsoft Office™, that are readily accessible to less sophisticated users. In the future, it may be desirable to port GDT to UNIX and related operating systems. This would require using an alternative database such as MySql™, Oracle™ or PostGres™. As experience with the program grows users may discover additional features that when implemented will further facilitate genomic research andbioinformatics.

## Availability and requirements

**Project name: **Genome Display Tool

**Project home page: **

**Operating system(s): **Windows 9x or higher

**Programming language: **Java

**Other requirements: **Java 1.4.1 or higher, MS-Access 2000 or higher

**License: **GNU GPL.

**Any restrictions to use by non-academics: **license needed

## Abbreviations

1. GDT – Genome Display Tool

2. COG – Clusters of Orthologous Groups

3. NCBI – National Center for Biotechnology Information

## Competing interests

An invention disclosure has been filed with the University of Houston.

## Authors' contributions

LV upgraded the original unpublished version of the software, modified it to meet additional requirements, tested it and wrote the first and subsequent draft of the paper, created the website and updated the user's manual. YL implemented the original version of the Gene Display Tool and generated the original users manual. GEF oversaw the project, developed the design requirements of the software, tested it, recognized the generality of the approach, and edited the paper and user manual.
